# P-1873. Telavancin Outcomes in Infections Treated with Outpatient Parenteral Antibiotic Therapy (OPAT)

**DOI:** 10.1093/ofid/ofae631.2034

**Published:** 2025-01-29

**Authors:** Brian S Metzger, Nikhil K Bhayani, Thomas K Sleweon, Rishi Battacharyya, Richard L Hengel, Kimberly A Couch, Theresa L Human, Emily A Durr, Jason M Makii, Lucinda J Van Anglen

**Affiliations:** Austin Infectious Disease Consultants, Austin, Texas; DFW Infectious Diseases, PLLC, Bedford, Texas; Infectious Disease Specialists, Highland, Indiana; Infectious Disease Associates, Sarasota, Florida; Atlanta ID Group, Atlanta, Georgia; Healix Infusion Therapy, LLC, Sugar Land, Texas; Cumberland Pharmaceuticals, Nashville, Tennessee; Cumberland Pharmaceuticals, Nashville, Tennessee; Cumberland Pharmaceuticals, Nashville, Tennessee; Healix Infusion Therapy, LLC, Sugar Land, Texas

## Abstract

**Background:**

Telavancin (TLV) is a lipoglycopeptide approved for the treatment of complicated skin and skin structure infection (cSSSI) and hospital acquired and ventilator associated bacterial pneumonia caused by susceptible Gram-positive bacteria. Data is lacking for published outcomes in OPAT. This study evaluated the use and treatment outcomes of TLV in patients receiving OPAT.

**Figure:**
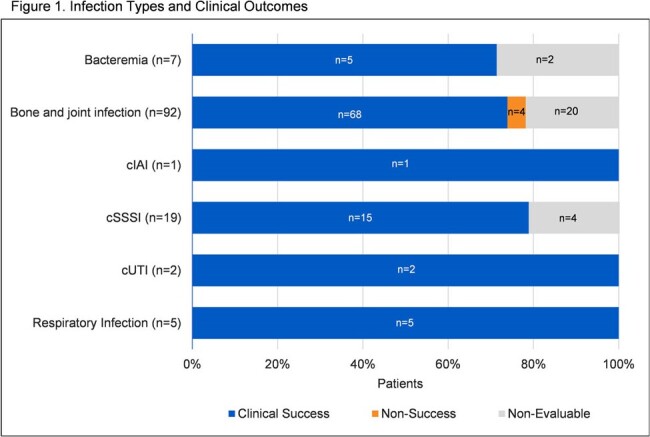

**Methods:**

Medical records of adult patients who received TLV during 2021-2023 in OPAT were queried. Demographics, diagnosis, therapy characteristics, laboratory and microbiologic data, adverse events (AEs), and clinical outcomes were collected. Patients were categorized at the completion of OPAT as clinical success (complete or partial resolution of signs and symptoms of infection without need for escalation of antimicrobials), non-success (persistent infection or discontinuation of TLV due to non-improvement), or non-evaluable (unable to determine clinical response to TLV). The microbiologically evaluable group (presence of Gram-positive bacterial culture data) was evaluated for microbiologic success (clearance of Gram-positive organism or improvement/resolution of infection) or microbiologic failure (persistent growth of a Gram-positive organism or non-improvement of infection). Descriptive statistics were used.

**Results:**

A total of 126 patients from 33 infusion centers were included. Median age was 57 (IQR: 47-64) years and 65% were male. Median TLV daily dose was 750 mg (8mg/kg/dose) and median duration of treatment was 28 days (IQR: 14-40). Gram-positive pathogens were documented in 74 (59%) of patients. Predominant infections included bone and joint (73%) and cSSSI (15%). Early TLV discontinuation occurred in 40 (32%) patients. Overall, 22% prematurely ended TLV due to an AE, most commonly nausea/vomiting (4%) and rash (4%). Of 100 patients evaluable for outcome, clinical success was achieved in 96 (96%) and non-success in 4 (4%). The clinical outcomes for each infection type are shown in Figure 1. Microbiologic success was achieved in 63 (95%) and 3 (5%) experienced microbiologic failure.

**Conclusion:**

TLV use in OPAT was associated with a high clinical success rate. TLV presents an effective option for treatment of Gram-positive infections of multiple etiologies in OPAT.

**Disclosures:**

Brian S. Metzger, MD, MPH, Cumberland Pharmaceuticals: Advisor/Consultant|Ferring Pharmaceuticals: Advisor/Consultant Nikhil K. Bhayani, MD, FIDSA, CorMedix: Advisor/Consultant|Cumberland Pharmaceuticals: Advisor/Consultant|Melinta: Advisor/Consultant|Paratek Pharmaceuticals: Advisor/Consultant Richard L. Hengel, MD, Gilead: Grant/Research Support Theresa L. Human, PharmD, Cumberland Pharmaceuticals: Employee Emily A. Durr, PharmD, Cumberland Pharmaceuticals: Employee Jason M. Makii, PharmD, Cumberland Pharmaceuticals: Employee Lucinda J. Van Anglen, PharmD, Cumberland Pharmaceuticals: Grant/Research Support|Ferring Pharmaceuticals: Grant/Research Support|Novartis Pharmaceuticals: Grant/Research Support|Takeda Pharmaceuticals: Grant/Research Support

